# Chaperoning STAT3/5 by Heat Shock Proteins: Interest of Their Targeting in Cancer Therapy

**DOI:** 10.3390/cancers12010021

**Published:** 2019-12-19

**Authors:** Gaëtan Jego, François Hermetet, François Girodon, Carmen Garrido

**Affiliations:** 1 INSERM, LNC UMR1231, team HSP-Pathies, University of Bourgogne Franche-Comté, F-21000 Dijon, France; francois.hermetet@u-bourgogne.fr (F.H.); francois.girodon@chu-dijon.fr (F.G.); 2 UFR des Sciences de Santé, University of Burgundy and Franche-Comté, F-21000 Dijon, France; 3 Haematology laboratory, Dijon University Hospital, F-21000 Dijon, France; 4 Centre Georges François Leclerc, 21000 Dijon, France

**Keywords:** heat shock proteins, chaperones, stabilization, targeted therapy

## Abstract

While cells from multicellular organisms are dependent upon exogenous signals for their survival, growth, and proliferation, commitment to a specific cell fate requires the correct folding and maturation of proteins, as well as the degradation of misfolded or aggregated proteins within the cell. This general control of protein quality involves the expression and the activity of molecular chaperones such as heat shock proteins (HSPs). HSPs, through their interaction with the STAT3/STAT5 transcription factor pathway, can be crucial both for the tumorigenic properties of cancer cells (cell proliferation, survival) and for the microenvironmental immune cell compartment (differentiation, activation, cytokine secretion) that contributes to immunosuppression, which, in turn, potentially promotes tumor progression. Understanding the contribution of chaperones such as HSP27, HSP70, HSP90, and HSP110 to the STAT3/5 signaling pathway has raised the possibility of targeting such HSPs to specifically restrain STAT3/5 oncogenic functions. In this review, we present how HSPs control STAT3 and STAT5 activation, and vice versa, how the STAT signaling pathways modulate HSP expression. We also discuss whether targeting HSPs is a valid therapeutic option and which HSP would be the best candidate for such a strategy.

## 1. Introduction to Heat Shock Proteins/Chaperones

Heat shock proteins (HSPs), also called stress proteins, are highly conserved molecular chaperones induced by a broad variety of exogenous or intracellular stresses, including chemotherapy. Based on their molecular weight, HSPs have been classified into five conserved families: HSP110 (also called HSPH), HSP90 (HSPC), HSP70 (HSPA), HSP60 (HSPD/E), and the small HSPs (HSPB). The expression of HSPs is mostly regulated by heat shock factor 1 (HSF1), which is able to translocate from the cytoplasm to the nucleus following stress to bind to a short, highly conserved DNA sequence known as a heat shock element (HSE) [1]. As cytoprotective proteins, HSPs participate in the correct folding, activity, transport, and stability of proteins [2], which are essential processes for cell survival. In physiological conditions, these proteins support neosynthesized proteins, favoring post-translational modification processes and protein folding. Otherwise, the functions attributed to them include the subcellular transport of their “client” proteins, or participation in certain signaling pathways. In response to stress, many partially denatured proteins accumulate and cluster together forming protein aggregates via the exposure of their hydrophobic residues. Some HSP proteins are then able to bind to these partially denatured proteins, thus preventing protein aggregation and favoring their correct folding. However, HSPs can also promote the elimination of these proteins by orienting them towards different degradation pathways, notably the ubiquitin–proteasome system, when correct folding is no longer possible. It has also been reported that HSP proteins are able to inhibit the intrinsic apoptotic (inhibition of apoptosome formation) and extrinsic (inhibition of signal transduction of death receptors) processes [3,4]. Interestingly, HSPs can also be secreted and act extracellularly via membrane receptors or within extracellular vesicles as a damage-associated molecular pattern and exhibit immune-cell dysregulation properties.

## 2. HSP Chaperones and Cancer

As cancer cells accumulate mutations and generate physiologically stressful conditions, they require a constitutively high level of HSPs for their survival and maintenance. In 2011, in order to simplify the complexity of this disease, researchers suggested that tumor development was organized around six essential alterations [5]. These major modifications include (i) self-sufficiency in growth signals; (ii) insensitivity to growth inhibition; (iii) tissue invasion and capacity to develop metastases; (iv) unlimited replication potential; (v) de novo angiogenesis; and (vi) inhibition of programmed cell death. Although the appearance of these changes is mainly linked to instability genomics, many studies have demonstrated the involvement of HSPs in these processes, indicating that these molecular chaperones have an oncogenic role. Comprehensive discussion about the oncogene-like functions of these different molecular chaperones and their participation in the progression of resistance to cancer treatment can be found in excellent recent reviews elsewhere [6,7]. Given the role of HSPs in cancer biology, these chaperones have also been suggested as potential therapeutic targets [3,8]. A number of these proteins have been correlated to cancer aggressiveness and/or cancer resistance to radiotherapy and adjuvant chemotherapy [9].

Targeting HSPs has emerged as a promising sensitization strategy in cancer therapy since HSPs have oncogene-like functions and mediate “non-oncogene addiction” of stressed tumor cells that must adapt to a hostile microenvironment. Except for one inhibitor of HSP27 (an antisense oligonucleotide in phase I/II) [10], all the HSP inhibitors used in clinical trials target HSP90 [11,12]. In this review, we mainly focused on the chaperones HSP90, HSP70, HSP110, and HSP27 and their regulation of protein misfolding and signaling in TYK2-STAT3/5 core cancer pathways, as well as the possibility of targeting such HSPs to specifically restrain STAT3/5 oncogenic functions. We also discuss the machinery behind the chaperones, which is becoming a major therapeutic target in cancer, and the emergence of promising HSP inhibitor-based drugs, which are currently being clinically tested or developed for cancer treatment (Table 1).

## 3. HSP90

### 3.1. HSP90 Structure and Functions

HSP90 (also known as HSPC) is one of the most abundant chaperones in eukaryotic cells in the absence of stress. HSP90 is critical for the operation of cellular machinery under physiological conditions through interactions with so-called “client” proteins. This is only achieved through the formation of a multimeric protein complex of cochaperones that binds to all three domains of HSP90. Hundreds of client proteins for HSP90 have been identified so far [73]. Many of these proteins are involved in essential cellular functions that promote cell growth, proliferation, cell survival, and immune responses. Most of these processes are also involved in cancer development. Three main groups of “client proteins” can be described for HSP90: first, the group of kinases represents the main group because HSP90 interacts with 60% of them [74]; second, the group of multiprotein complexes for which HSP90 promotes assembly [75]; and third, the group of ligands that HSP90 stabilizes with their receptors. It is difficult to identify new client proteins because HSP90 does not bind particular sequences. In contrast, the interaction seems to be based on the overall structural instability of the client proteins [74,76]. Among the client proteins, here we focus on the kinases and receptor tyrosine kinases involved in the STAT3/5 signaling pathway.

### 3.2. HSP90 and Nonfusion Protein Kinases

#### 3.2.1. Jak Kinases

The mammalian family of Janus kinases (JAKs) is composed of 4 members: JAK1, JAK2, JAK3, and Tyrosine kinase 2 (Tyk2). This family is the main activator of STAT proteins. JAK regulation by HSP90 was discovered by studying the effect of HSP90 inhibitors on the type I and II Interferon (IFN) signaling. In several cell lines, this treatment suppressed the expression of multiple IFN-γ-induced genes and decreased IFN-γ-induced STAT1 phosphorylation on Tyr-701, required for dimerization, and on Ser-727, required for transcription factor activation. As JAK1/2 were known to be the protein kinases responsible for STAT1 phosphorylation, Shang et al. investigated the effect of HSP90 inhibitors on JAK1/2. They showed that HSP90 inhibition led to the proteasome-mediated degradation of JAK1/2. Further they showed that JAK1 interacted with HSP90 (and the CDC37 cochaperone [77]), and that both interactions were destabilized by HSP90 inhibitors [78]. As overactivation or constitutive JAK1/2 signaling promotes cell proliferation and survival in a variety of solid tumors and leukemia [79,80], this discovery paved the way for the identification of the critical role of HSP90 in the aberrant JAK/STAT signaling pathway. In particular, an activating point mutation in JAK2 (*JAK2*^V617F^) was described as being highly frequent in chronic myeloproliferative neoplasms (MPN) that promote disease progression [81,82,83,84].

Despite this activating mutation, HSP90 inhibition in cell lines homozygous for *JAK2*^V617F^ reduced total and phospho-JAK2, and subsequently cell viability [85]. In vivo experiments in a mouse model of MPN confirmed the efficacy of HSP90 targeting because treatment with the HSP90 inhibitor PU-H71 resulted in significant reductions in disease parameters and better chances of survival [49]. Furthermore, combined treatment that included HSP90 inhibitors and JAK2 inhibitors induced a greater depletion of the signaling proteins than a single inhibitor alone, and synergistically induced apoptosis in human primary CD34(+) MPN cells harboring JAK2^V617F^ [50]. Therefore, HSP90 interaction with JAK2 is not altered by activated mutations, but instead could be used as a therapeutic target. This point is of great value, as mutations within the JAK2 kinase domain that confer resistance across a panel of JAK inhibitors have been described (G935R, Y931C, and E864K). Fortunately, genetic resistance to JAK2 enzymatic inhibitors can be overcome by HSP90 inhibitors, which still promote the degradation of both wild-type and mutant JAK2 [60]. Recently, results from a phase II clinical trial with the HSP90 inhibitor AUY922 (Novartis, transferred to Vernalis) have been published and have demonstrated a clinical response in five out of seven patients with MPN [58]. This response correlated with a reduction in overall levels of JAK2, pYSTAT3, and pYSTAT5. Unfortunately, most patients experienced severe adverse effects due to the toxicity of the inhibitor, a phenomenon that has already been observed with other HSP90 inhibitors.

#### 3.2.2. Src Kinases

The members of the Src family of nonreceptor tyrosine kinases (Src, Fyn, Yes, Blk, Yrk, Rak, Fgr, Hck, Lck, Srm, and Lyn) are implicated in numerous important functions in eukaryotic cells. They control proliferation, survival, and differentiation, therefore playing a critical role in many cancer types [86]. Src members can activate STAT3 directly and synergize with JAK family tyrosine kinase action [87]. Among the family members, c-Src has been linked to cancer development [88]. The viral homolog of c-Src kinase, v-Src (from the *Rous sarcoma* virus), has a constitutive kinase activity and was the first discovered oncogene [89,90]. Both homologs bind to the HSP90/CDC37 complex but with striking differences. HSP90, which binds weakly and transiently to c-Src, binds strongly to v-Src, which appears to be its strongest client protein [91,92,93]. Accordingly, v-Src kinase activity depends strongly on HSP90 [74,94]. Recently, Boczek et al. provided more insight by determining the influence of HSP90 isoforms α and β on purified c-Src and v-Src activity. They have shown that HSP90 does not affect c-Src activity in vitro, whereas v-Src activity was increased two-fold when human HSP90β (but not HSP90α) was added to the experimental setting. HSP90β also stabilized v-Src at high temperatures when it would be inactive otherwise. Until recently, the mechanism behind this striking difference was unknown [76,95]. To solve this issue, Bolcek et al. generated an Src mutant that mimics the oncogenic v-Src kinase activity (c-src3MΔC). This mutant exhibited a more extended activation loop (A-loop) (usually present in an open form during wild-type Src active state to allow substrate binding). The A-loop from c-Src3MΔC is also less stable in comparison with the wild-type Src. Consequently, the c-Src3MΔC is conformationally uncontrolled, which enhances its interaction with HSP90 and suggests this could be a more general mechanism for the interaction between HSP90 and oncogenic kinases than the presence of a general client sequence motif. Indeed, HSP90 potentially interacts more strongly with structurally extended kinases, a frequent state observed upon activating mutations. Interestingly, a very similar mechanism needed to aid the initial folding of immature kinases such as c-Src, which is furtive is this case, governs the binding of HSP90 to conformationally unstable but mature kinases like v-Src. In this context, CDC37 appears to bind to parts of the unfolded kinase first (which might be considered as an independent kinase binding unit), partly unfolding it further before HSP90 clamps around the CDC37/kinase complex [96]. Other Src family members, like Lck^Y505F^ and HCK^499F^, are probably stabilized by the same mechanism [97,98].

#### 3.2.3. ACK1

Another nonreceptor tyrosine kinase, activated CDC42-associated kinase-1 (ACK1), catalyzes the phosphorylation of STAT1, STAT3, and STAT5. HSP90 interacts with ACK1 [99] and is necessary for the phosphorylation of STAT1 in transformed kidney cells and STAT3 in primary lung adenocarcinoma by ACK1 [61].

#### 3.2.4. BRAF

The activated serine/threonine kinase BRAF mutant is a main driver of melanoma growth and progression [100] and is a HSP90 client protein [74,101]. Inhibition of HSP90 by AT13387 delays the emergence of resistance to BRAF inhibitors [62]. A recent phase I dose escalation clinical trial in melanoma has shown that another HSP90 inhibitor (XL888) in combination with a specific anti BRAF inhibitor (vemurafenib) has clinical activity in patients with advanced BRAF^V600^-mutant melanoma, with a tolerable side effect profile [65].

### 3.3. HSP90 and Fusion Protein Kinases

#### 3.3.1. BCR-ABL

Chronic myeloid leukemia (CML) is driven by the BCR-ABL fusion oncoprotein [102], which is involved, among other pathways, in the transcriptional regulation of STAT3 [103,104] and STAT5 [105,106]. In this context, the BCR-ABL/STAT3/STAT5 signaling pathway is mainly involved in tumor-initiating stem cell maintenance [107]. BCR-ABL is a HSP90 client protein that is destabilized by HSP90 inhibition, which leads to cell death [56]. In CML cells, BCR-ABL forms a high molecular weight network with JAK2, STAT3, and AKT. This network pushes disease progression, but could also be its Achilles’ heel. Indeed, HSP90 directly binds to this signaling network, and its inhibition breaks the whole network apart [56]. As for other targeted therapies, resistance to BCR-ABL tyrosine kinase inhibitors can develop during the course of the treatment because of acquired *BCR-ABL* mutations. Hopefully, combination therapies involving HSP90 inhibitors and anti-JAK2 may overcome this resistance [57].

We are still unsure of how this mechanism of action can be extended to the interaction with other oncogenic mature kinases, but an important process for protein stabilization by HSP90 and CDC37 has been uncovered.

#### 3.3.2. EML4-ALK

The echinoderm microtubule-associated protein-like 4-anaplastic lymphoma kinase (EML4-ALK) fusion gene is an oncogenic driver in about 5% of patients with non-small cell lung cancer (NSCLC). It is also an HSP90 client protein (one of the most sensitive), which is very rapidly degraded upon exposure to HSP90 inhibitors [40]. These results prompted the initiation of numerous clinical trials reviewed elsewhere [41,108]. Yet despite encouraging results, clinical response was weak, and so the development of HSP90 inhibitors was halted for NSCLC.

### 3.4. HSP90 and ErbB Family of Receptor Tyrosine Kinase (RTK)

STAT3 and STAT5 are also known to be phosphorylated by several receptor tyrosine kinases (RTK), such as the ErbB family, IGF-1R, or FGFR [109], most of which are HSP90 client proteins. Interestingly, they are also activated or captured for signal transduction by those RTK without phosphorylation [110]. Given their membrane localization, these RTK must go through a complex process of folding, maturation, and membrane insertion that requires significant chaperone cooperation. This is particularly true for mutated RTK, which is frequently observed in cancers. For instance, ErbB2 stability and maturation is regulated by its binding to HSP90 through its cytoplasmic tail [111] and is ATP dependent [112].

Accordingly, HSP90 inhibition leads to RTK destabilization and absence of STAT3 activation in different models of cancer [47,66,67]. Many drugs have been developed to inhibit mutated or rearranged RTK, but despite early success, most patients develop resistance and eventually relapse [113]. The strong HSP90/EGFR interaction has then been used to propose an alternative therapeutic strategy combining an HSP90 inhibitor with an EGFR inhibitor. Interestingly, this combination (with the EGFR inhibitor erlotinib) resulted in prolonged animal survival in nonmutated and erlotinib-resistant models [67,68,70,71,114,115].

### 3.5. STAT3/5 and HSP90

The STAT3/5 signaling pathway is also regulated downstream from the tyrosine kinases and RTK. Indeed, HSP90 is found within the cytosol, directly bound to dimers of STAT3 or STAT5 via its N-terminal regions [116]. However, in contrast to its role in TK or RTK folding and stabilization, HSP90 is not required for STAT3/5 maturation or total protein levels. They are therefore nonclassical HSP90 client proteins. The chaperone would rather change STAT conformation to ease the phosphorylation process and/or, once phosphorylated, maintain this active state for a prolonged period of time. Moulick et al. have suggested this pattern in chronic myeloid leukemia [69]. They showed that HSP90 directly binds to active pYSTAT5 (Tyr694), but not to inactive STAT5, and that pYSTAT5 acquires a conformation that is more susceptible to trypsine cleavage in the presence of HSP90. HSP90/STAT3 also protects pYSTAT3 from dephosphorylation by the phosphatase SHP-1 in gastric cancer cells. Luteolin (3,4,5,7-tetrahydroxyflavone), a natural flavonoid present in fruits and vegetables, inhibits STAT3 activation by disrupting the association of HSP90 to STAT3, which allows it to interact with SHP-1 [117].

In order to function as a transcription factor, STAT3/5 needs to translocate into the nucleus and form a stable interaction with DNA. In this context, as suggested by Longshaw et al., HSP90 appears to play a specific role in association with the cochaperone HOP [118]. They have shown that the depletion of HOP decreased the nuclear localization of STAT3. Although it is not yet clear how HSP90 promotes STAT3/5 nuclear shuttling, it may involve the capacity of HSP90 to form molecular complexes with specific carriers, like importins alpha [119], that can transport STAT3 to the nucleus. This scheme would mimic what has been described for other molecular complexes implicating HSP90, such as the glucocorticoid receptor [120] or PKCZeta [121]. After entering the nucleus, HSP90 seems to promote STAT3/5 transcriptional activity as STAT3/5 interaction with promoters of target genes is enhanced by the presence of HSP90. Indeed, the STAT3/5 complexes and HSP90 have been shown to colocalize in MYC and in CCND2 promoters [69]. Furthermore, nuclear hormone receptors form multiprotein complexes with STAT3 and STAT5 [122,123], which together with HSPs could contribute to chromatin landscaping [124].

**Figure 1:**
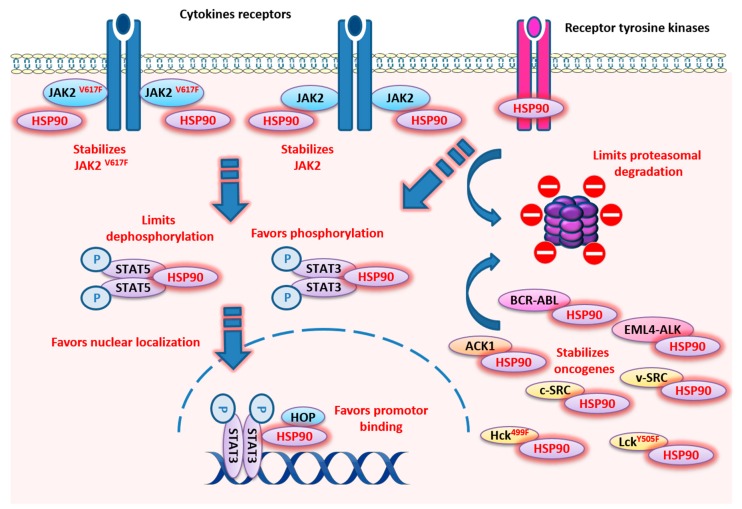
Localization and described functions of HSP90 within the STAT3 and STAT5 signaling pathways. HSP90 promotes those pathways through direct interaction with STAT3 or STAT5 dimers and favors their phosphorylation, nuclear localization, and promoter binding, but HSP90 also limits dephosphorylation and proteasomal degradation. Upstream of STAT3/STAT5 activation, HSP90 stabilizes several kinases, like JAK2, JAK2^V617F^, c-Src, v-Src, ACK1, BCR-ABL, EML4-ALK, Lck^Y505F^, and HCK^499F^, and several receptor tyrosine kinases, such as the ErbB family.

**Figure 2:**
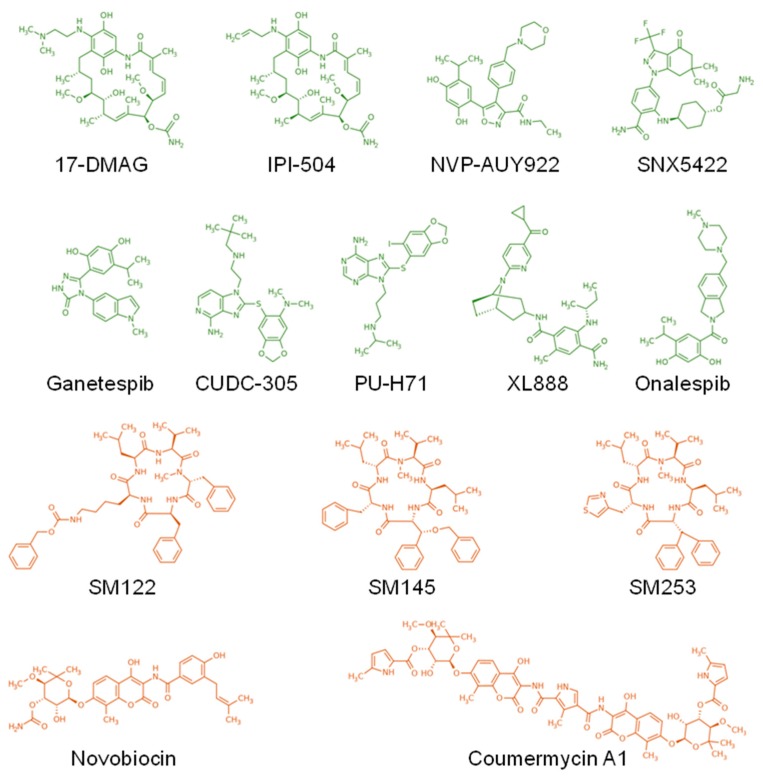
Structure of the main HSP90 inhibitors. The inhibitors targeting the ATP binding site at the N-terminus and the C-terminus of HSP90 are depicted in green and orange, respectively.

In conclusion, HSP90 appears to be a key chaperone for the STAT3/5 pathway. It operates at all levels of message transmission, from interaction with RTK in the cytoplasmic membrane, to the interaction with multiple kinases in the cytosol, and to favoring active STAT3/5 localization and binding of target genes the nucleus (Figure 1). This role is also central in the pathological overactivation of STAT signaling where HSP90 favors oncogenic proteins (Figure 1), promoting the development of several inhibitors for cancer treatment (Table 1, Figure 2). However so far, most clinical trials have yielded mixed results and frequent side effects that precluded the broad utilization of these treatments.

## 4. HSP27

### 4.1. HSP27 Structure

HSP27 (27 kDa), also known as HSPB1, belongs to the small HSP family. In contrast to other HSPs, it is an ATP-independent chaperone [125]. HSP27 shows a highly dynamic process of oligomerization that transforms proteins from dimers to large oligomers, which can culminate at 1000 kDa. This state of oligomerization dictates the affinity of HSP27 towards the proteins to be chaperoned, given that the multimer form is the most binding-competent state [126].

Four phosphorylation sites (S15, S78, S82 and T143) in the N-terminal domain regulate the assembly of oligomers [127]. Phosphorylation promotes the formation of small oligomers, while dephosphorylation promotes the formation of large oligomers [128]. Stressors such as anticancer agents, hydrogen peroxide, mitogens, inflammatory cytokines (TNF-α, IL-1b, etc.), and kinases (p38 MAPK, p70RSK, PKB, PKC, PKD and PKG) can promote HSP27 phosphorylation [129]. However, we have also shown that oligomerization can occur independently of phosphorylation through cell–cell contact, as observed in confluent cultures in vitro or solid tumors in vivo [130].

### 4.2. HSP27 Functions

HSP27 chaperone functions have been less described than higher molecular weight HSPs like HSP70 and HSP90. However, HSP27’s main function as a chaperone is to stabilize denatured or aggregated proteins to bring them to a soluble and stable form [131,132].

HSP27 plays an important role and has been associated with poor prognosis in many cancers (for a recent review, see [127]). For instance, Rocchi et al. described the promotion of the STAT3 signaling pathway in prostate tumors [133]. They showed that HSP27 directly interacts with STAT3 and that total STAT3 levels correlated directly with HSP27 levels. Furthermore, the cytoprotective effect of HSP27 was attenuated by STAT3-reduced expression, underlying the importance of this pathway in prostate cancer [133]. HSP27 is also involved in the process of prostate cancer metastasis through the promotion of IL-6-mediated epithelial-to-mesenchymal transition (EMT), because reduced HSP27 expression decreases cell migration and invasion [134]. In the absence of HSP27, IL-6-induced phosphorylation of STAT3 is reduced, but not total STAT3 content. This inhibition leads naturally to reduced STAT3 nuclear localization and binding to the *TWIST* gene, which codes for a key EMT transcription factor.

Beside cancer development, HSP27 regulation of STAT3 has recently been implicated in placental implantation. HSP27 is indeed expressed during placenta formation and in the first two trimesters of pregnancy [135,136]. In particular, HSP27 is highly expressed during the differentiation of cytotrophoblast cells and extravillous trophoblast cells, and its silencing was found to significantly reduce total STAT3. Interestingly, the phosphorylation state of STAT3 was not altered in the absence of HSP27 in placental explants, suggesting a role in protein protection from proteasomal degradation [137]. This could be explained by the fact that STAT3 and STAT5 have a relatively low thermodynamic stability as isolated proteins and are thus more prone to aggregation, which would be limited by HSP27 [124]. Given the importance of STAT3 in embryonic development (STAT3 knock-out mice have a lethal embryonic phenotype) [138], this finding revealed the critical role of HSP27 in this process.

There has been little study of the state of HSP27 phosphorylation, and consequently the state of oligomerization, required for STAT3/5 binding, despite the fact that therapeutic targeting of specific kinases would logically impact HSP27 functions. We only know, from one study of prostate cancer, that IL-6 stimulation leads to HSP27 phosphorylation and correlates with the EMT, suggesting the phosphorylated form is required for STAT3 activation [134]. Other STAT family members are HSP27 client proteins, like STAT2 (a STAT family member involved in viral or interferon responses), which was also shown to be degraded upon HSP27 knockdown in Hela cells [139]. However, this process was reversed by proteasome inhibition. However, STAT3 and 5 were not or were only weakly reduced in this particular tumor cell line. Again, the discrepancy between tumoral contexts or cell lines could come from differences in HSP27 phosphorylation or oligomerization status.

As stated previously, HSP27/STAT3 interaction occurs also in nontumoral contexts. In normal liver cells under a high fat diet, the phosphorylated form of HSP27 stimulates autophagy and lipid droplet clearance through interaction with STAT3. In this particular situation, no STAT3 activation by HSP27 is described, but rather the disruption of STAT3/PKR complexes, facilitating PKR and eIF2α mediated autophagy [140]. These data suggest that dimers and multiprotein complexes can be displaced by the action of phospho-HSP27 on different binding partners, including STAT3, and this can also mediate critical cellular physiological processes.

Upstream from STAT3/5 activation, JAK2 plays a major role that can be modulated by HSP27 [141]. In the specific context of thrombopoietin- and JAK2^V617F^-induced myelofibrosis (a chronic degenerative disorder of the hematopoietic system associated with the aberrant activation of the JAK/STAT pathway) [142], our team has recently shown that HSP27 interacts directly with JAK2/STAT5, stabilizing the complex. Neither total JAK2 nor STAT5 protein levels were affected, but we found that the state of phosphorylation of STAT5 (Tyr694) by JAK2 was HSP27 dependent. We demonstrated that HSP27, through interaction with STAT5, physically prevented its dephosphorylation by the phosphatase SHP2 in those cells.

**Figure 3:**
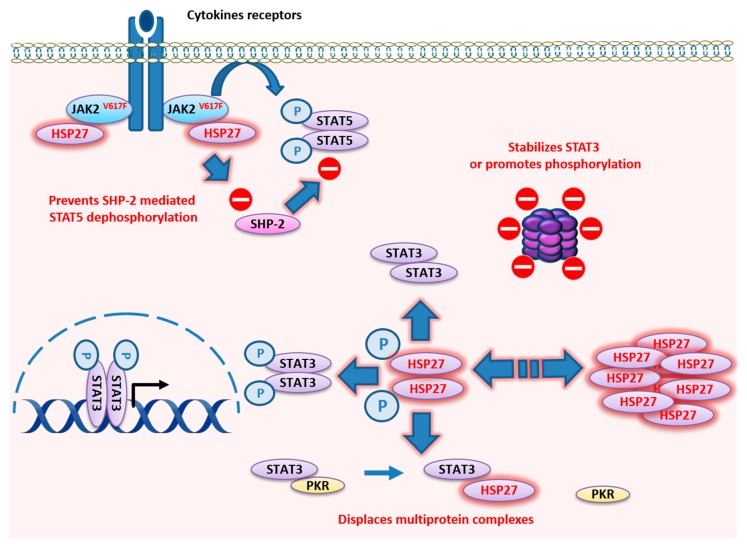
Localization and described functions of HSP27 within the STAT3 and STAT5 signaling pathways. HSP27 state of oligomerization varies dynamically to modulate its binding capacity to target proteins in a context-dependent way. HSP27 directly interacts with pYSTAT3 and total STAT3 to promote stabilization and phosphorylation. HSP27 directly binds to JAK2^V617F^/STAT5 complexes to prevent STAT5 dephosphorylation by SHP-2 in MPN. HSP27 also displaces multiprotein complexes like the STAT3/PKR complex.

In conclusion, HSP27 plays a very important role in STAT3/5 signaling, both in contexts of tumor development and others such as placenta development. In contrast with HSP90, whose functions depend on the client proteins, HSP27′s different functions (protein stability, phosphorylation, disruption of complex of proteins, etc.) may rely on the phosphorylation and oligomerization status of the chaperone (Figure 3). The general vision is mainly dichotomous: on one side there are large phosphorylated oligomers, and on the other nonphosphorylated dimers. This simplistic description does not reflect the reality of cellular dynamics. Future studies will be needed to specify the proportion of each HSP27 oligomer within the cells and the associated function.

## 5. HSP110

### 5.1. HSP110 Structure

The 110 kDa heat shock protein (HSP110), also known as HSP105 or HSPH1, belongs to the members of the HSPH family. Although it appears to be distinct from other HSPs (HSP27, HSP40, HSP70, and HSP90) because of its molecular weight and the specificity of certain sequences, HSP110 is a member of the family of HSP70 proteins [143,144]. Until quite recently, HSP110 was considered as a mere nucleotide exchange factor of HSP70. However, it is now well established that HSP110 is able to act as an unfolding chaperone on its own using ATP hydrolysis to lead to conversion of stable misfolded polypeptide substrates into natively refolded products, even when HSP70 is not present [145]. In an ATP-independent manner, HSP110 also has the antiaggregating properties of unfolded or misfolded proteins.

### 5.2. HSP110 Functions

**Figure 4:**
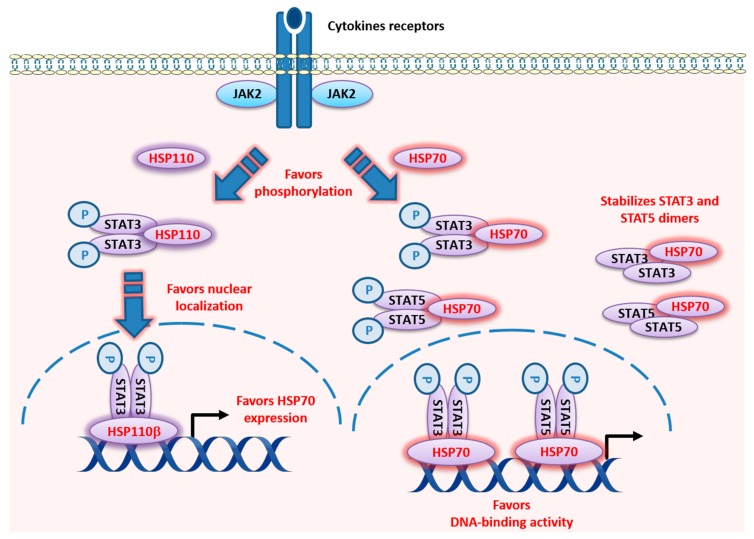
Localization and described functions of HSP110 and HSP70 within the STAT3 and STAT5 signaling pathways. HSP110 directly binds to STAT3 in the cytosol and favors its phosphorylation through JAK2, and, through this mechanism, participates in the promotion of cell proliferation. HSP110α and HSP110β localize to the cytoplasm and nucleus of cells, respectively. HSP110β induces the expression of HSP70 in mammalian cells. Overexpression of HSP110β stimulated the phosphorylation of STAT3 (Tyr705) and its translocation to the nucleus. STAT3 binds to the sequence of the HSP70 promoter at the level of a sequence (−206 to −187 base pair) whose mutation abrogated the activation of the HSP110β-mediated HSP70 promoter. HSP70 directly interacts with STAT3 and STAT5. It favors STAT3 phosphorylation and activity, and STAT5 levels, phosphorylation, and activity.

In contrast to other chaperones, like HSP90 or HSP70, little is known about the cellular and extracellular functions of HSP110. It is a ubiquitous and conserved chaperone with antiaggregation capabilities that act in synergy with the refolding activity of HSP70, which contributes to efficient protein homeostasis [145,146]. HSP110 expression is induced by a wide array of stress including hyperthermia, ethanol, oxidative stress, recovery from anoxia (i.e., reperfusion injury), some anticancer drugs, and inflammation. HSP110 is approximately four-fold more efficient at binding and stabilizing denatured protein substrates than HSP70 [147]. Due to its strong chaperone (or holder) function, HSP110 is a very good antigen carrier. It is therefore used in vaccine formulations [148] as a recombinant chaperone vaccine for antigen-targeted cancer immunotherapy. These vaccines have generated robust antigen-specific T lymphocyte responses in different preclinical cancer models [149]. Two forms of HSP110 exist: HSP110α and an alternatively spliced form called HSP110β, which contains 43 fewer amino acids [150]. While HSP110α is constitutively expressed in the cytoplasm of cells and can be induced in stressful conditions such as heat shock, HSP110β is strictly heat-inducible and specifically localized in the nucleus (Figure 4) [151]. Beyond the different localization of these two forms of HSP110, their differential roles remain unclear. Like other HSP proteins, the expression of HSP110 can be induced by a number of physical or chemical sources of stress and depends on the heat shock factor 1 (HSF1) transcription factor. Moreover, the presence STAT3 on the HSP110 promoter has been recently reported in humans, suggesting its regulation by STAT3 [152]. Conversely, HSP110β can induce HSP70 expression through STAT3 in mammalian cells (Figure 4) [153]. It is now clearly established that HSP110 favors several signaling pathways, including the Wnt/β-Catenin, MyD88/TLR, and STAT3 pathways [154,155].

Concerning the STAT signaling pathway, we have demonstrated that HSP110 directly binds to STAT3 and favors its phosphorylation (Tyr705) by JAK2 in the cytosol, thereby promoting cell proliferation (Figure 4) [156]. Colon cancer cells in which HSP110 has been shRNA-mediated and knocked down hardly proliferate, but proliferation is reactivated by the re-expression of HSP110 in these cells. Tumors from patients with high levels of HSP110 show high STAT3 phosphorylation levels and strong expression of proliferation markers [154,156]. Therefore, both the protein homeostasis function and the role of HSP110 on proliferative pathways may explain why this protein is linked to aggressive tumors. We suggest that HSP110 expression could be a surrogate prognostic marker and a potential therapeutic target, particularly for treatment of carcinomas, particularly colorectal cancer, for which there is strong evidence.

**Figure 5:**
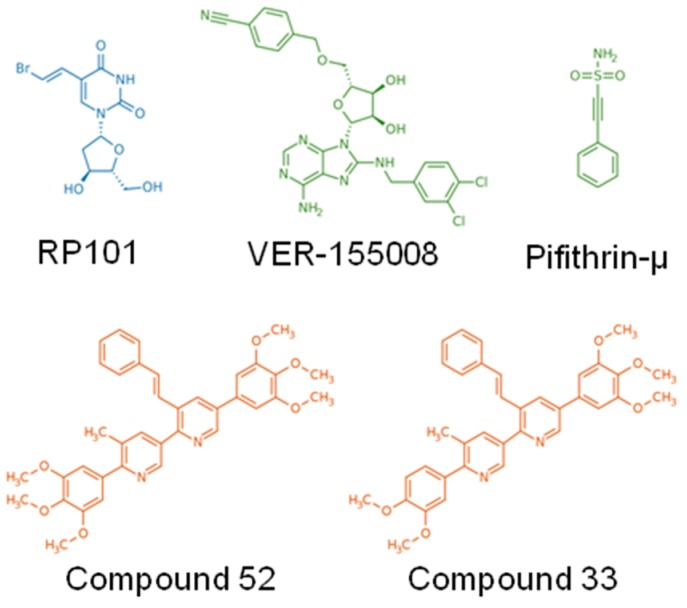
Structure of the main small-molecule inhibitors of HSP27 (blue), HSP70 (Green), and HSP110 (Orange). RP101 can inhibit HSP27 function via direct binding to Phe29 and Phe33. VER-155008 binds to the ATP-binding site at the N-terminus of HSP70. Pifithrin-µ inhibits specifically function of HSP70 via direct binding to its substrate binding domain. Compounds 52 and 33 bind to the ATP binding site at the N-terminus of HSP110.

Given the emerging role of HSP110 in cancer and its role on STAT3 in particular, we selected two foldamers upon screening of a chemical library based on their ability to inhibit and block recombinant HSP110-mediated antiaggregation activity and to disrupt HSP110–STAT3 interaction [72]. These compounds, named 33 and 52 (Table 1, Figure 5), inhibit HSP110 chaperone function and colorectal cancer growth in vitro and in vivo [72]. Altogether these results confirm the interest of targeting HSP110, at least in colorectal cancers, and probably in other types of cancer, such as B-cell lymphoma.

Although HSPs are generally considered intracellular proteins, we now know that HSP110 can also be released to act extracellularly like HSP27, 70, and 90 [157,158,159]. The release of HSP110 from human intestinal epithelial cells has also been described, suggesting a role in the physiological process of epithelial renewal [160]. More recently, we have demonstrated that HSP110, like other HSPs, can be secreted by cancer cells and is abundantly observed in the cancer microenvironment [161]. Interestingly, extracellular HSP110 affects macrophage differentiation/polarization by favoring a protumor, anti-inflammatory profile and the formation of tumor-associated macrophages (TAMs), which are associated with immune suppression. Furthermore, we found a correlation between the level of extracellular HSP110 and the number of TAMs in patient biopsies [161], suggesting that the effect of extracellular HSP110 function on macrophages may also contribute to the poor outcomes that are associated with HSP110 expression.

## 6. HSP70

### 6.1. HSP70 Structure

Stress-inducible HSP70 (also called HSP72 or HSP1A, HSPA1B) is another member of the HSP superfamily that has emerged as a viable and very promising target for development of antitumor drugs to combat various forms of cancer [162,163,164]. As the most ubiquitous stress-inducible chaperone, HSP70 exhibits numerous chaperone functions that are critical for both the folding and proteasomal degradation of misfolded proteins. It thus participates in cellular protein quality control systems, leading to cell homeostasis and survival during stress conditions. The human HSP70 protein family consists of at least 13 members, including stress-induced HSP70 and the heat shock cognate protein 70 (HSC70) [165]. The chaperone mechanism of HSP70 has been extensively studied [166]. HSP70 associates with misfolded proteins in a manner controlled by its ATPase cycle and cochaperones such as HSP40, BAG family proteins, HIP, HOP, and HSPBP1 [165].

### 6.2. HSP70 Functions

Based on immunoprecipitation analysis from normal rat kidney interstitial fibroblast (NRK-49F) cells, HSP70 has been shown to directly interact with multiple STAT proteins, including STAT3/5 (Figure 4). This interaction increased when cells were stressed by exposure to advanced glycation end product [167]. Although the data are still limited, studies show that manipulating HSP70 expression or activity affects STAT protein activity within cells. HSP70 (HSP701A/B) knock down using siRNA further decreased constitutive STAT3 activity in an acute myeloid leukemia cell line (HEL) treated with arsenic trioxide and the HSP90 inhibitor 17-DMAG [168]. In addition, increasing HSP70 activity with administration of geranylgeranylacetone before and three days after intracerebral hemorrhage resulted in increased STAT3 and AKT phosphorylation and also cerebral levels of eNOS, which are collectively associated with preserved cerebral blood flow, decreased neuronal cell death, and improved functional recovery in rats [169]. Uchida et al. suggested that the mechanism for HSP70 induction through geranylgeranylacetone may be the result of geranylgeranylacetone-induced induction of protein kinase C [170].

It is worth noting that upregulation of HSP70 could stimulate cell proliferation through the control of tyrosine kinase functions. In cancer cells derived from chronic myeloid leukemia cells, BCR-ABL tyrosine kinase activity results in the phosphorylation and activation of AKT, and in the phosphorylation and DNA-binding activity of STAT5, which leads in turn to an increase in the expression of antiapoptotic protein Bcl-xL [171]. Interestingly, while increased expression of HSP70 results in the upregulation of STAT5 level and activity, inhibition of the BCR-ABL tyrosine kinase activity with imatinib or inhibition of PI-3K activity with wortmannin both result in a decrease in HSP70 expression and STAT5 activity. Thus, uncontrolled cell signaling may result in the transcription of HSP70, which in turn regulates the level and activity of STAT5 (Figure 4) [171]. Besides the intracellular role of HSP70 as a survival factor that promotes tumorigenesis [172], it is now known that HSP70 also regulates diverse immunoregulatory activities such as antigen cross presentation [173,174], dendritic cell maturation [175,176], and natural killer cell [177,178] and myeloid-derived suppressor cell [179] activities, by acting extracellularly as a cytokine [180,181]. We demonstrated that membrane-associated HSP70 is found extracellularly in tumor-derived exosomes and that it restrained tumor immune surveillance by promoting myeloid-derived suppressor cell functions in both mice and humans. Interestingly, tumor-derived exosomes harboring HSP70 were found to mediate the suppressive activity of the myeloid-derived suppressor cells via activation of STAT3 and ERK [179].

## 7. Regulation Mechanisms of HSF/HSPs by JAK/STAT Signaling: A Feedback Loop?

### 7.1. HSF and SOCS Regulation

The activation of the JAK family and the subsequent STAT signaling is regulated by the family of suppressors of cytokine signaling (SOCS) proteins. This system aims to protect organisms from permanent and/or overstimulation that could lead, for instance, to severe systemic inflammations mediated by IFN-γ signaling [182]. Conversely, a deficit in SOCS expression could play a pivotal role in the development and progression of cancers [183]. Expression of SOCS3, which has been shown to inhibit JAK1, JAK2, and TYK2 [184], is frequently reduced in cancer cells, thereby leading to a growth advantage. The effect of HSF1 and HSP on SOCS3 expression changes depending on the tissue and whether the context is normal or tumoral. In nontumoral microglia cells, the activation of HSF1 by paeoniflorin induced an indirect increase of SOCS3 expression mediated by HSP70 production and autocrine action [185]. Conversely, it was recently shown that HSF1 could directly bind to the SOCS3 promoter region and inhibit the transcriptional activity of its promoter [186]. This reinforces the role of HSF1 as a transcription factor for many genes not related to heat shock response [3]. SOCS3 repression of expression can also be mediated by HSPs, as seen in chronic lymphocytic leukemia. In this context, HSP90 inhibition by 17-DMAG induces the expression of SOCS3 through the activation of the p38 MAPK signaling [34]. This regulation is probably a specific to tumors, as normal B cells were not found to upregulate SOCS3 expression upon HSP90 inhibition, and their migration was not affected [34].

### 7.2. HSF/HSPs and JAK/STAT

The regulation of the STAT signaling pathway by HSPs has been largely described, but in a positive feedback loop STAT3/5 has been shown to regulate HSPs. Little is known about HSP regulation apart from the fundamental role of HSF. The STAT3/5 pathway modulates expression of HSP27, HSP70, HSP90, and HSP110 when faced with different stressful stimuli [187]. Among those, mild heat shock is a type of stress (that was used to first describe the function of HSPs) during which the induction of HSPs prevents cell death from intense heat shock that otherwise would have been lethal. This thermotolerance is also accompanied by STAT3 phosphorylation. Inhibition of STAT3 activation by STAT3 inhibitors AG490 or static partially suppresses thermotolerance and HSP110 expression, but not HSP70 and HSP27 [188]. This result underlines a particular role for HSP110 in the process, which is in line with its capacity to interact with α-tubulin. This mechanism could therefore protect microtubules from severe heat shock [189]. Besides STAT3, STAT1 is also involved in HSP70 and HSP90 regulation, particularly after IFN-γ activation [190,191] or under heat shock [192]. Together with HSF1, STAT1 is recruited to the first intron of the HSP90β gene to favor the recruitment of a chromatin-remodeling complex, leading to enhanced HSP90β expression.

Other types of stress, such as hypoxic stress (a common phenomenon in a majority of tumors), are strong inducers of HSP90α expression. In these conditions, STAT5 is one of the transcription factors that regulate HSP90α, and hypoxia increases the binding of STAT5 to the HSP90α promoter [193]. When the STAT signaling pathway is constitutively active in tumors, like in breast and colon cancer, STAT3 has also been shown to transcriptionally induce HSP27 expression [194]. Though less documented, this mechanism also exists in hematological malignancies like Burkitt’s lymphoma, where pharmacological inhibition of STAT3 by AG490 downregulates HSP70 expression [195].

## 8. Conclusions

We have reviewed here the multiple roles of HSPs in the STAT3/5 activation network. From receptor tyrosine kinases to promoter binding of STAT3/5 target genes, HSPs appear to use several mechanisms to control this pathway. Among the various HSPs, HSP90 has a central role in the cellular machinery and is one of the most abundant cytosolic proteins. This role is clearly exposed when we describe both kinases and STAT proteins as HSP90 client proteins. The current literature points to the important role of HSP90/STAT3/STAT5 in cancer growth and the ability to thwart chemotherapy [3,196,197,198].

This discovery has prompted the development of drugs specifically targeting HSP90 and the subsequent initiation of clinical trials for cancer patients (Table 1, Figure 2). Almost all of the trials have reported clinical responses, confirming the relevance of this strategy. However, adverse side-effects were also frequent due to the inherent toxicity of HSP90 inhibitors. This could be explained by the number of client proteins that are simultaneously chaperoned by HSP90, which obviously endangers critical physiological processes in normal cells. A strategic question emerges from this observation: should we continue searching for more specific, less toxic drugs? Or should we limit studies to fundamental science to uncover new biological mechanisms? Other HSPs are gaining interest in the scientific community. In particular, HSP110 is a newly discovered player in the field of cancer aggressiveness that controls the STAT3 pathway in colon cancers and in B-cell lymphomas. Our team recently identified the first HSP110 inhibitors (Table 1, Figure 5) that block interaction with STAT3 and that effectively limit colon cancer progression in mouse models; we plan to bring these to the clinic within the next few years. No toxicity has been demonstrated so far, and we believe this new type of inhibitor is promising for cancers whose poor prognosis is associated with HSP110/pYSTAT3 expression. Both intra- and extracellular HSP70, which belong to a related HSP family, favor STAT3/5 phosphorylation by JAK2. This dual action is of particular therapeutic interest since targeting HSP70 would simultaneously blunt the macrophage-mediated immunosuppression and block the intratumoral growth signal. To reach the goal of bringing HSP70 inhibitors to the clinic, we have identified peptide aptamers that bind to the peptide-binding domain of HSP70. This has not been an easy task since HSP70, contrary to HSP90, is not a “druggable” protein. Despite this limitation, our HSP70 inhibitors are specific and have proven effective in xenograft models of colon cancer. Of course, further studies will be needed before clinical trials can be initiated. Finally, HSP27 has recently been found to play new roles in myeloproliferative neoplasms. In this disease, which is driven by the JAK2^V617F^/STAT5 pathway, HSP27 inhibition destabilizes the protein complex and limits disease related myelofibrosis. Furthermore, in contrast to HSP90 inhibitors, the HSP27 inhibitor does not induce the compensatory expression of other HSPs that usually account for resistance to treatment. Targeting HSP27 would therefore be an alternative to the failed HSP90 therapy, which also targeted JAK2^V617F^. An oligonucleotide antisense of second generation (OGX427) is currently under clinical evaluation (Table 1).

Furthermore, small molecules targeting STAT3/5 have been identified as enhancing protein degradation. The inhibition of STAT pathways is therefore likely highly amenable to HSP inhibition and presents a potential synergistic therapeutic strategy (Table 1).

In conclusion, we show in this review that the STAT3/5 pathways rely on multiple HSPs under physiological and pathological conditions. Targeting various members of the HSP family, alone or in combination, will probably improve the inhibition of this central pathway and should foster the development of new, more specific and less toxic HSP inhibitors to complete the existing therapeutic arsenal.

## Figures and Tables

**Table 1:** Summary of the main strategies for HSP inhibition.

Inhibitor		Study Type	Cancer Model	Ref.
Name	Nature/Structure			
**Target: HSP27**				
Apatorsen (OGX-427)	2nd generation 2’-methoxyethyl-modified ASOs	in vitro/preclinical	Prostate*,* Ovary	[13,14]
		clinical trial (phase I)	CRPC, Breast, Ovary, Lung, Bladder	[10]
		in vitro/preclinical	Pancreatic, NSCLC	[15,16]
		clinical trial (phase II)	Stage IV non-squamous NSCLC	[17]
3-arylethynyltriazolyl ribonucleoside	ASOs	in vitro	Pancreatic	[18]
ASOs-Hsp27	ASOs	in vitro	Lymphoma	[19]
RP101 (Brivudine)	Uridine derivative and nucleoside analog	in vitro/preclinical/clinical	Pancreatic	[20]
**Target: HSP70**				
Pifithrin-µ (PFTµ, PES)	Drug-like small molecule	in vitro	AML, ALL, Primary AML blasts	[21,22]
VER-155008	ATP-derivative inhibitor	in vitro	Breast, Colon, Prostatic, Myeloma	[23,24,25]
A17/A8	Peptide aptamer	in vitro/preclinical	Cervix (HeLa cells), Melanoma	[26]
ADD70	Peptide aptamer	in vitro/preclinical	Rat colon carcinoma, Mouse melanoma	[27]
cmHsp70.1	Antibody	preclinical	Colorectal	[28]
Hsp70-peptide targeted NK based adoptive immunotherapy	A specific amino acid sequence (TKD) of Hsp70	clinical trials (phase I/II)	NSCLC (and colon cancer) patients with ex vivo Hsp70 peptide activated, autologous NK	[29]
**Target: HSP90**				
Radicicol	natural product isolated from the fungus *Monosporium bonorden*	in vitro	CML	[30]
17-AAG; 17-DMAG	Derivative of the antibiotic geldanamycin	in vitro/preclinical	Breast, Brain, Medulloblastoma	[31,32,33]
17-DMAG		in vitro	CLL	[34]
IPI-504 (retaspimycin)	Water-soluble derivate of 17-AAG	in vitro/preclinical	Breast, Pancreatic, Metastatic gastrointestinal stromal tumor	[35,36,37,38,39]
		in vitro/preclinical	NSCLC	[40]
IPI-504, AUY922 Ganetespib, Onalespib	-	clinical trials (phase I–III)	NSCLC Breast, Ovary, Colon	[41]
Novobiocin	Aminocoumarin antibiotic, produced by the actinomycete *Streptomyces nivens*	in vitro/preclinical	Leukemia*, *Prostate	[42,43,44]
Panaxynol	Natural pesticide and fatty alcohol	in vitro/preclinical	Lung	[45]
Ganetespib (STA-9090)	Synthetic, non-geldanamycin, small molecule inhibitor	preclinical	Thyroid	[46]
		in vitro	Breast	[47]
BIIB021 (CNF2024)	Orally available, fully synthetic purine scaffold, small molecule inhibitor	in vitro/preclinical	Blood malignancies, Solid tumors	[48]
PU-H71	Non-ansamycin, purine scaffold inhibitor	preclinical	mouse models of the MPN PV and ET	[49]
			MPN	[50]
NVP_AUY922 (AUY922)	Esorcinylic isoxazole amide, second-generation non-geldanamycin inhibitor	in vitro/preclinical	Gastric, Small cell lung, Thyroid	[51,52,53,54,55]
		in vitro	32D mouse hematopoietic cells expressing wild-type BCR-ABL (b3a2, 32Dp210) and mutant BCR-ABL imatinib-resistant cell lines	[56]
		in vitro/preclinical	Drug-resistant chronic myelogenous leukemia	[57]
		clinical trial (phase II)	Myeloproliferative neoplasms	[58]
		clinical trials (phase I/II)	EGFR-mutant lung cancer with acquired resistance to epidermal growth factor receptor tyrosine kinase inhibitors	[59]
AUY922, HSP990, PU-H71	-	in vitro/preclinical	Leukemia	[60]
Onalespib (AT13387)	second-generation, non-ansamycin inhibitor	in vitro	Transformed kidney cells, primary lung adenocarcinoma	[61]
		in vitro/preclinical	Melanoma	[62]
		in vitro/preclinical	NSCLC	[63]
		in vitro/preclinical	NSCLC	[64]
XL888	Orally available inhibitor with high selectivity for HSP90α and HSP90β	clinical trial (phase I)	Melanoma	[65]
SNX2112 SNX5422	Orally bioavailable, synthetic, small molecule inhibitors that competitively bind to HSP90α, HSP90β, Grp94 and Trap-1	in vitro/preclinical	Head and neck squamous cell carcinoma	[66]
			NSCLC	[67]
CUDC-305, Ganetespib CH5164840, WK88-1 17-DMAG	-	preclinical	NSCLC	[68,69,70,71]
**Target: HSP110**				
Foldamers 33 and 52	Protein–protein interaction inhibitors, based on pyridyl scaffolds mimicking α-helix	in vitro/preclinical	Colorectal	[72]

ALL: acute lymphoblastic leukemia; AML: acute myeloid leukemia; ASOs: antisense oligonucleotides; CLL: chronic lymphocytic leukemia; CML: chronic myelogenous leukemia; CRPC: castration-resistant prostate cancer; EGFR: epidermal growth factor receptor; ET: essential thrombocytosis; MPN: myeloproliferative neoplasm; NK: natural killer cells; NSCLC: non-small cell lung cancer; PES: 2-phenylethynesulfonamide; PV: polycythemia vera.
